# A newly identified linear epitope on non-RBD region of SARS-CoV-2 spike protein improves the serological detection rate of COVID-19 patients

**DOI:** 10.1186/s12866-021-02241-y

**Published:** 2021-06-26

**Authors:** Yunwen Zhang, Zhengrong Yang, Sicheng Tian, Baisheng Li, Tiejian Feng, Jianfan He, Min Jiang, Xiujuan Tang, Shujiang Mei, Hao Li, Yifan Zhong, Guilian Li, Mingyuan Tang, Sijing Liu, Tian Tang, Chuan Wang, Xiaohui Wang

**Affiliations:** 1 West China School of Public Health and West China Fourth Hospital, Sichuan University, Chengdu, PR China; 2 Food Safety Monitoring and Risk Assessment Key Laboratory of Sichuan Province, Department of Public Health Laboratory Sciences, West China School of Public Health, Sichuan University, Chengdu, PR China; 3 Shenzhen Center for Disease Control and Prevention, Shenzhen, China; 4 Guangdong Provincial Center for Disease Control and Prevention, Guangdong, China

**Keywords:** SARS-CoV-2, Spike protein, Epitopes, Humoral immunity, Antibody

## Abstract

**Background:**

Serological test is helpful in confirming and tracking infectious diseases in large population with the advantage of fast and convenience. Using the specific epitope peptides identified from the whole antigen as the detection antigen is sensitive and relatively economical. The development of epitope peptide-based detection kits for COVID-19 patients requires comprehensive information about epitope peptides. But the data on B cell epitope of SARS-CoV-2 spike protein is still limited. More importantly, there is a lack of serological data on the peptides in the population. In this study, we aimed to identify the B cell epitope peptides of spike protein and detect the reactivity in serum samples, for further providing data support for their subsequent serological applications.

**Results:**

Two B cell linear epitopes, P104 and P82, located in non-RBD region of SARS-CoV-2 S protein were identified by indirect ELISA screening of an overlapping peptide library of the S protein with COVID-19 patients’ convalescent serum. And the peptides were verified by testing with 165 serum samples. P104 has not been reported previously; P82 is contained in peptide S21P2 reported before. The positive reaction rates of epitope peptides S14P5 and S21P2, the two non-RBD region epitopes identified by Poh et al., and P82 and P104 were 77.0%, 73.9%, 61.2% and 30.3%, respectively, for 165 convalescent sera, including 30 asymptomatic patients. Although P104 had the lowest positive rate for total patients (30.3%), it exhibited slight advantage for detection of asymptomatic infections (36.7%). Combination of epitopes significantly improved the positive reaction rate. Among all combination patterns, (S14P5 + S21P2 + P104) pattern exhibited the highest positive reaction rate for all patients (92.7%), as well as for asymptomatic infections (86.7%), confirming the feasibility of P104 as supplementary antigen for serological detection. In addition, we analyzed the correlation between epitopes with neutralizing antibody, but only S14P5 had a medium positive correlation with neutralizing antibody titre (r_s_ = 0.510, *P* < 0.01).

**Conclusion:**

Our research proved that epitopes on non-RBD region are of value in serological detection especially when combination more than one epitope, thus providing serological reaction information about the four epitopes, which has valuable references for their usage.

**Supplementary Information:**

The online version contains supplementary material available at 10.1186/s12866-021-02241-y.

## Background

The coronavirus disease 2019 (COVID-19) outbreak began in Wuhan, China, in December 2019. In March 2020, the World Health Organization (WHO) announced that COVID-19 had become a global pandemic. As of 3 May 2021, there have been over 150 million confirmed cases of COVID-19, including over 3 million deaths, reported by WHO (https://www.who.int/). Besides nucleic acid detection, antibody detection has also been paid more and more attention in COVID-19 confirmation [1, 2]. The detection of specific antibody was helpful for confirmation of suspected cases and the identification of asymptomatic infection [3, 4]. In addition, antibody monitoring can assist in the evaluation of vaccine immune level and disease progression, provide necessary laboratory data on evaluating the disease transmission in populations and regions.

The spike (S) protein is the main detection target antigen of SARS-CoV-2. The specific antibody induced by S protein, especially the neutralizing antibody against receptor binding domain (RBD), plays a main role in inhibiting viral infections [5]. Both anti-S antibody and anti-RBD antibody are positively correlated with neutralizing antibody, and also positively correlated with disease severity [6, 7]. The anti-S antibody can indirectly reflect the neutralizing activity of sera and the severity of illness. Thus, the S protein is an ideal antigen for detection. The epitope peptides-based ELISA has economic benefits and convenience [8, 9]. Therefore, we conducted serological test to identify B cell epitope peptides of S protein, provided accurate antibody binding sequences and valuable antigen targets for the development of the serological test kit.

On January 8, 2020, the Shenzhen Centre for Disease Control and Prevention confirmed the first case of COVID-19 in Shenzhen [10]. Shenzhen is one of the biggest cities in China, with a population over 10 million. After the outbreak of COVID-19 in China, strict isolation and testing measures were implemented to effectively control the spread of the epidemic. We began to collect the convalescent serum of COVID-19 patients, design and synthesize peptides library of S protein from March 2020, just shortly after the outbreak of COVID-19 in China, to screen and identify B cell linear epitopes on S protein. In this study, total of 165 serum samples of COVID-19 patients (including those without symptoms) in Shenzhen discharged after March 5 were collected. Through an indirect ELISA between the overlapping peptide library of the SARS-CoV-2 S protein and the convalescent serum, two linear epitopes, P104 and P82, specifically recognized by the convalescent serum immunoglobulin G (IgG) of COVID-19 patients. P82 is contained in the epitope S21P2, which reported by Poh et al. in June [11]. We then synthesized the epitope peptides S14P5 and S21P2 identified by Poh et al., analyzed the reactivity of the four epitope peptides with 165 convalescent serum samples. Furthermore, the RBD-IgG, RBD-total antibodies (RBD-Ab) and neutralizing antibody titre of serum were determined and the correlations of the antibody with four epitopes, age groups and disease severity were analyzed as well. Compared with other studies, our research used more convalescent serum samples to verify the reactivity and obtained more comprehensive data on epitope peptides.

## Results

### Characteristics of 165 discharged COVID-19 patients

Among the 165 patients, there were 87 women (52.7%) and 78 men (47.3%); the median age was 33 years old (4 to 86 years old). In total, 30 patients (18.2%) were asymptomatic, 22 (13.3%) had mild disease, 106 (64.2%) had moderate disease, and 7 (4.2%) had severe disease. In this study, there was only one critically ill patient. To facilitate group analysis, this patient was included in the severe group. The age groups were divided into minor group (0–18 years old), the young and middle-aged group (19–60 years old) and the elder group (61–90 years old) (Table 1).

**Table 1:** Demographics and clinical characteristics of COVID-19 patients

Characteristic	All patients (*n* = 165)	Clinical types			
		Asymptomatic (*n* = 30,18.2%)	Mild (*n* = 22,13.3%)	Moderate (*n* = 106, 64.2%)	Severe (*n* = 7, 4.2%)
Sex (n, %)					
Male	78 (47.3)	12 (7.3)	12 (7.3)	52 (31.5)	2 (1.2)
Female	87 (52.7)	18 (10.9)	10 (6.1)	54 (32.7)	5 (3.0)
Age (median)	33	27	23	36	57
Age group (n, %)					
0 ~ 18	22 (13.3)	16 (9.7)	5 (3.0)	0 (0.0)	1 (0.6)
19 ~ 60	125 (75.6)	14 (8.5)	17 (10.3)	90 (54.5)	4 (2.4)
61 ~ 90	18 (10.9)	0 (0.0)	0 (0.0)	16 (9.7)	2 (1.2)

### RBD-IgG, RBD-Ab and neutralizing antibody titre in COVID-19 convalescent serum

**Fig. 1:**
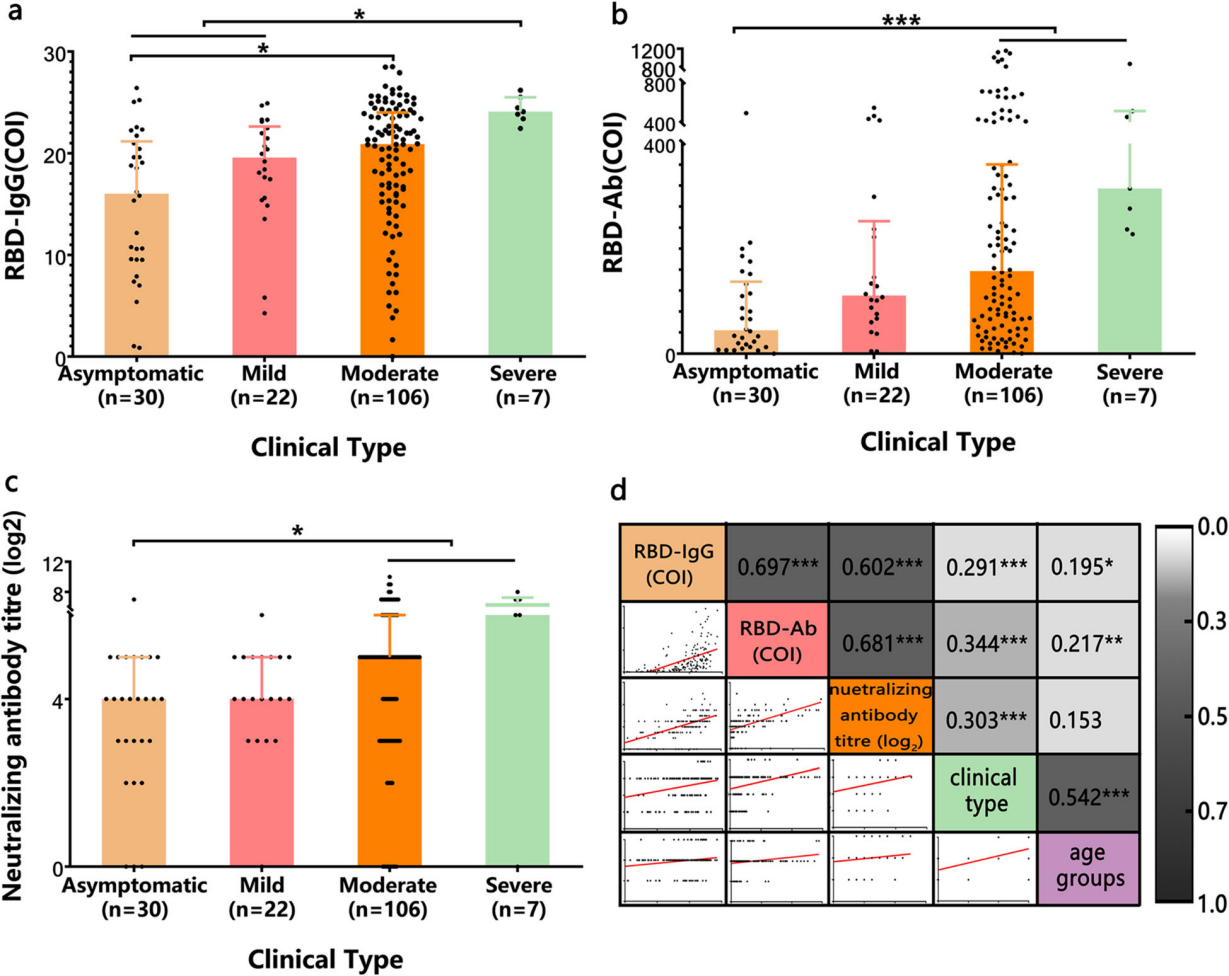
RBD-IgG, RBD-Ab and neutralizing antibody titre of COVID-19 discharged patients’ convalescent sera. **a-c** RBD-IgG (COI) **(a)**, RBD-Ab (COI) **(b)** and neutralizing antibody titre **(c)** of patients’ sera with different clinical types. Cutoff index (COI) value = the sample detection value/cutoff value. Each set of data was represented by the median (interquartile range). Each black dot represented the antibody detection value of each serum sample. **d** Correlation analysis among the RBD-IgG, RBD-Ab, neutralizing antibody titre, the disease severity and age groups. The figures showed the scatter plots, Spearman correlation coefficients and the red lines were trend lines

The results of the antibody analyses showed positive rates for RBD-IgG and RBD-Ab both were 98.8% (163/165). One patient from the asymptomatic infection group was negative for RBD-IgG and RBD-Ab, and two moderate patients were negative for RBD-IgG or RBD-Ab respectively. The RBD-IgG median (interquartile range, IQR) cutoff index (COI = sample detection value/ cutoff value) of convalescent COVID-19 sera was 20.43 (8.39), and the RBD-Ab median (IQR) COI was 128.39 (267.52). (Fig. 1a, b). The positive rate of neutralizing antibody was 86% (129/150), and the median (IQR) titre was 1:32 (1:24). (Fig. 1c). RBD-IgG and RBD-Ab showed moderately strong correlations with neutralizing antibody titre (Fig. 1d, r_s_ = 0.602, *P* < 0.01, r_s_ = 0.681, *P* < 0.01). RBD-IgG, RBD-Ab and neutralizing antibody were all positively correlated with disease severity (Fig. 1d). There was no difference in antibody level in the man and woman. Among age groups, the elderly had higher RBD-IgG levels than the minor (*P* < 0.05). And there was a weak correlation between antibody with age groups (RBD-IgG vs age groups: r_s_ = 0.195, *P* < 0.05. RBD-Ab vs age groups r_s_ = 0.217, *P* < 0.01). The disease severity was positively correlated with age groups (r_s_ = 0.542, *P* < 0.001).

### Dentification of B cell linear epitopes of the SARS-CoV-2 S protein

**Fig. 2:**
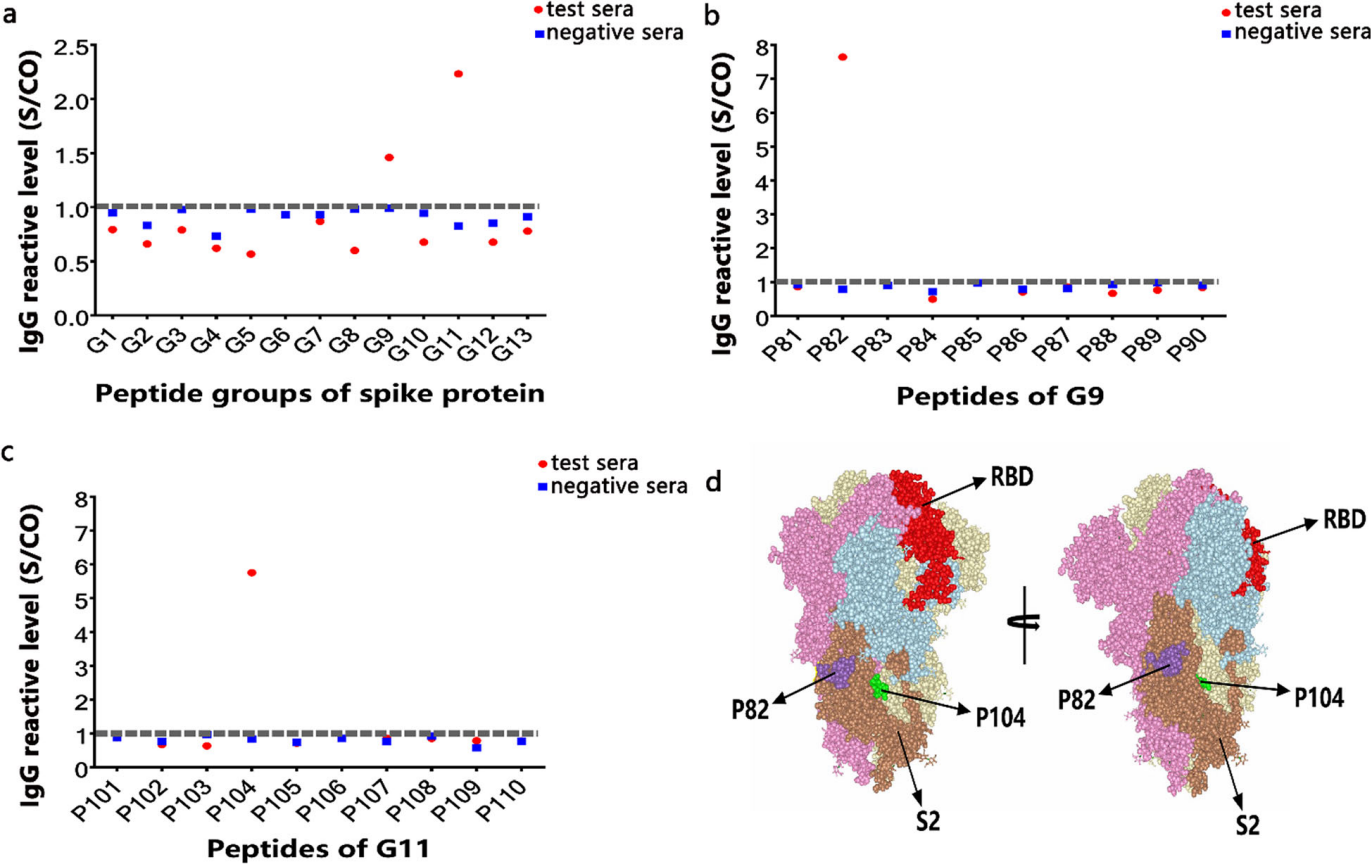
The identification of B cell linear epitopes in SARS-CoV-2 spike protein. **a** Each peptide group was tested with the test sera from twenty patients by indirect ELISA to screen the positive peptide groups (G9 and G11). **b-c** Performing indirect ELISA tests for each peptide contained in the selected positive peptide groups G9 and G11 with test sera to screen for the positive peptides (P82 and P104). Twenty convalescent serum samples randomly selected from the cohort that included fourteen moderate patients, three mild patients and three asymptomatic infections were pooled as test sera. Twenty serum samples of healthy people were pooled as negative sera. RBD of spike protein served as positive antigen control (data were not shown). The cutoff value was calculated as the mean + 3 × standard deviations (SD) of negative sera. The S/CO value (sample average OD_450 nm–630 nm_ value/cutoff value) of test sera was greater than 1 was considered to be a positive response. The red dots represented the S/CO values of test sera to peptides. The blue squares represented the S/CO values of negative sera to peptides. The grey dotted line indicated 1. Red dots above the grey line were considered to be a positive response. **d** The localization of two positive epitope peptides in spike protein. The 3D structure of SARS-CoV-2 spike protein was obtained from the NCBI website (PDB ID:6VXX). The image was processed through the NCBI website. The three monomers of spike protein were represented respectively as blue, pink, and grey. The positions of RBD, P82, P104 and S2 subunit in one monomer were distinguished by red, purple, green, brown and indicated by arrows

The overlapping peptides library of the S protein and the convalescent sera of COVID-19 patients were used to conduct indirect ELISA. In the first round of screening, the G9 and G11 peptide groups both had positive response values (Fig. 2a). Each peptide in G9 and G11 was then tested one-by-one with convalescent sera, and two B cell linear epitopes, P82 (811KPSKRSFIEDLLFNK825) and P104 (1031ECVLGQSKRVDFCGK1045), were identified by convalescent serum IgG (Fig. 2b, c, Supplementary Table 1). P82 is completely contained in the B cell epitope S21P2 reported by Poh et al. [4]; Poh et al. also identified another B cell epitope, S14P5 (Supplementary Table 1). P104 is a new epitope that partially overlaps between the epitope sequence predicted by Ahmed et al. (IEDB ID: 462 AATKMSECVLGQSKRVD, ID: 53202 RASANLAATKMSECVLG) [5]. Positioning P82 and P104 in the 3D structure of the S protein (PDB ID: 6VXX) showed that both P82 and P104 located in S protein subunit S2. In terms of spatial location, P82 is located on the surface of the protein and P104 in a groove on the surface (Fig. 2d).

### Positive reaction rates of epitope peptides S14P5, S21P2, P82 and P104 with convalescent serum

**Fig. 3:**
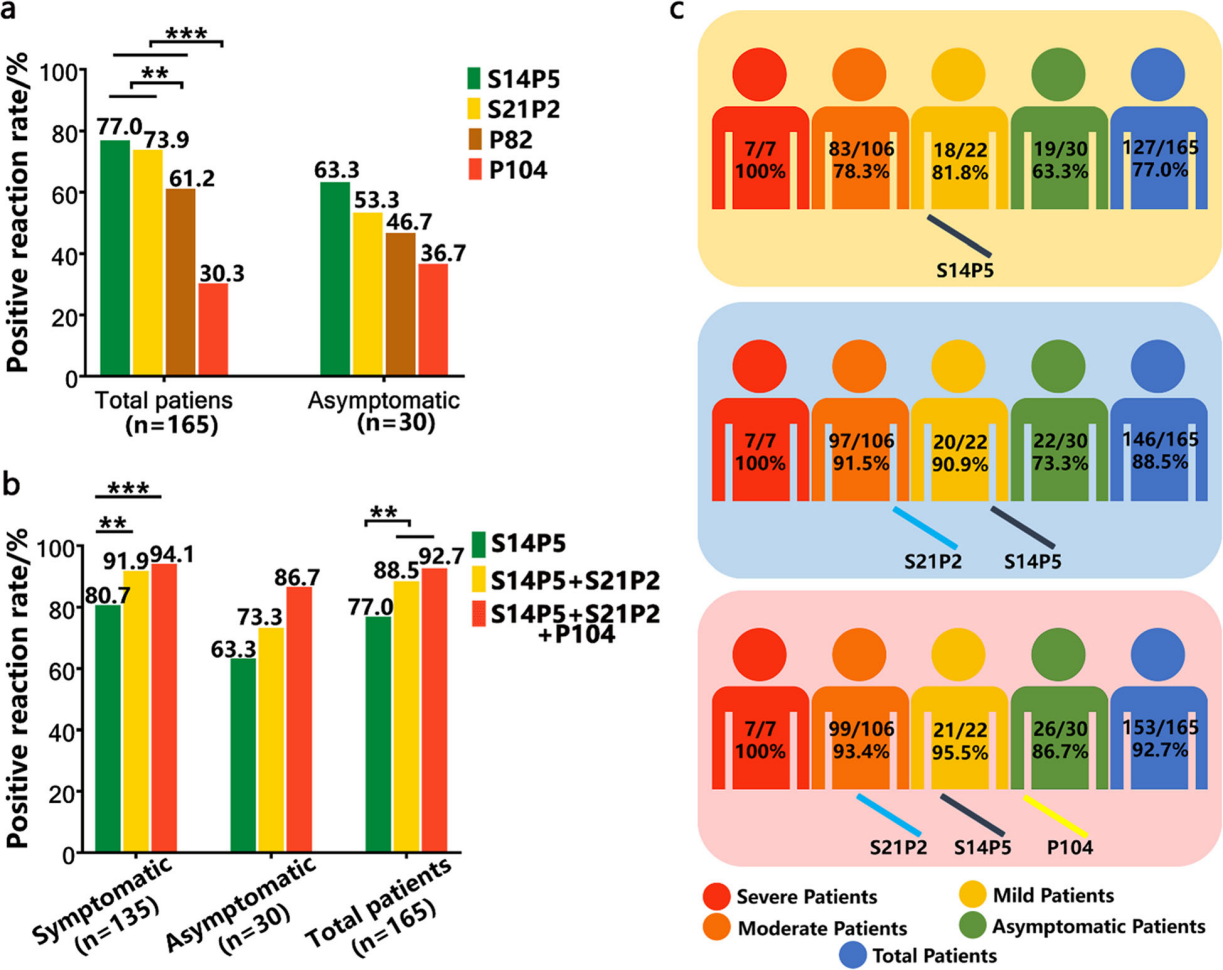
The positive reaction rates of epitope peptide S21P2, S14P5, P82 and P104 in convalescent sera of COVID-19 patients. Each convalescent serum sample of COVID-19 patients was tested with each epitope peptide by indirect ELISA. **a** The positive reaction rates of peptides S21P2, S14P5, P82 and P104 in total COVID-19 patients and asymptomatic infections. **b** Analyses of the positive reaction rates of different peptide combinations. S14P5, S14P5 + S21P2 and S14P5 + S21P2 + P104 were the best combinations with the highest overall positive reaction rate in peptide combinations respectively contained the single, two or the three epitope peptides. As long as the serum sample was positive for any one of the epitope peptides in combination, the sample was considered to be positive for the epitope peptide combination. **c** The positive reaction rates in different clinical types detected by different peptide combinations

Each convalescent serum from 165 COVID-19 patients was tested with the four epitope peptides (S14P5, S21P2, P82 and P104) by indirect ELISA. The overall positive reaction rates were 77.0%, 73.9%, 61.2% and 30.3%, respectively. The epitope peptides S14P5, S21P2 and P82 showed higher positive reaction rates, significantly different from P104 (*P* < 0.001). The positive reaction rates for the four epitope peptides with convalescent sera from 30 asymptomatic patients were 63.3%, 53.3%, 46.7%, and 36.7%, respectively (Fig. 3a, *P* = 0.209). The positive reaction rates of different epitope combination patterns were further compared in the symptomatic and asymptomatic patients. As long as the serum was positive to any one of the peptides in the combination, it was considered to be positive to the epitope peptide combination. The (S12P2 + S14P5) combination exhibited the highest positive reaction rate among combinations contained two epitopes; the (S12P2 + S14P5 + P104) pattern had the highest positive reaction rate among combinations contained three epitopes and its rate was same as four epitopes combination (Fig. 3b). Compared with single epitope peptide detection, the combined use of epitope peptides significantly increased the positive reaction rate in symptomatic patients (94.1%). It was surprised that combination use of epitope P104 increased positive reactive rate from 73.3% to 86.7% in asymptomatic patients. P104 single peptide had a poor overall positive reaction rate, but we noticed that 7 patients’ sera showed positive with P104 while the rest of epitopes cannot be recognized. Among the 7 patients, four of them were asymptomatic (Fig. 3c).

### Serum reactivity and antibody correlation analyses

**Fig. 4:**
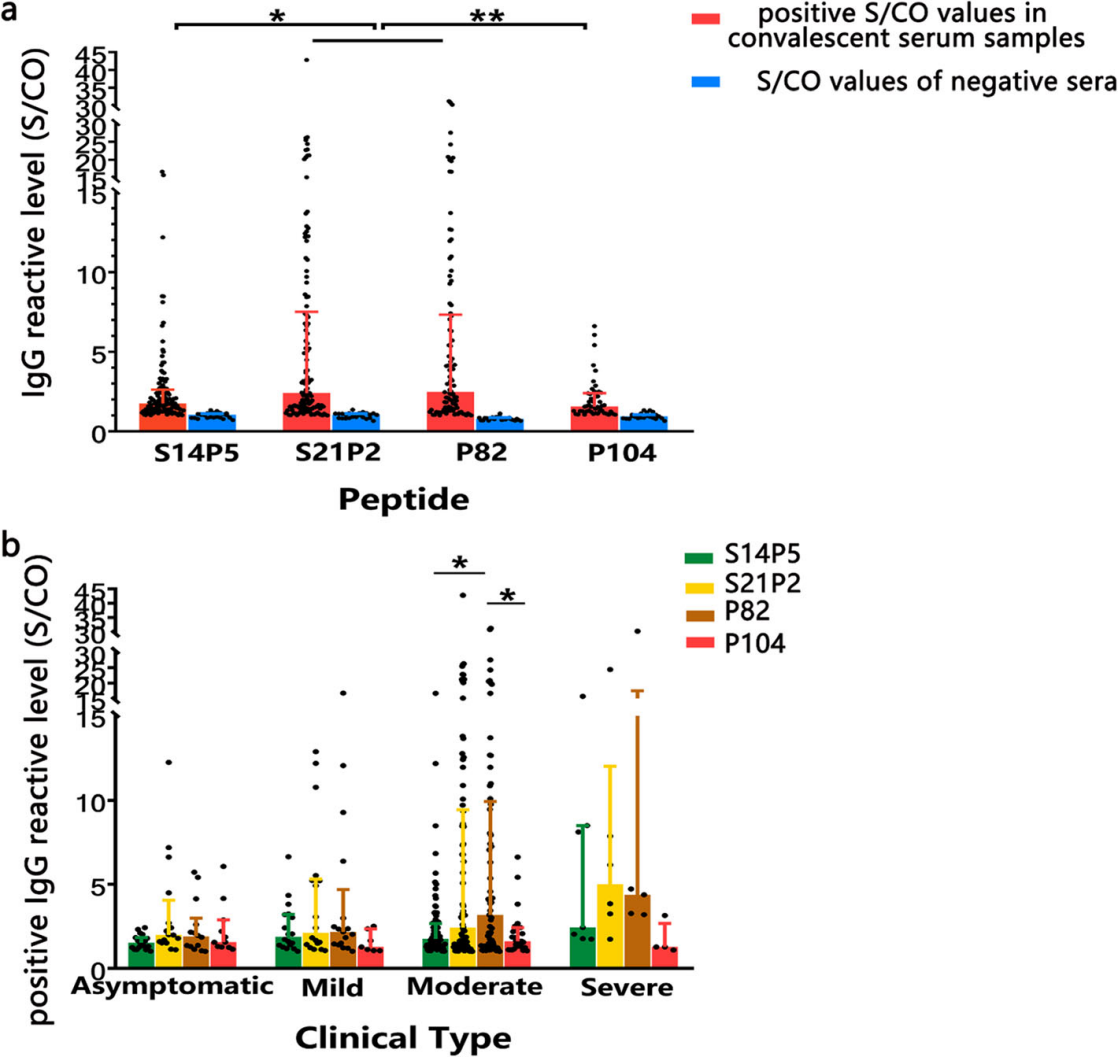
IgG reactive level (S/CO values) of epitope peptides. **a** The positive IgG reactive level (S/CO values) in convalescent serum samples and the S/CO values of negative sera to four epitope peptides respectively. **b** Positive IgG S/CO values of four epitope peptides with different clinical type patients. Each set of data was represented by the median (interquartile range)

The median (IQR) of positive IgG S/CO values for the convalescent sera from 165 COVID-19 patients with epitope peptides S14P5, S21P2, P82 and P104 were 1.7531 (1.3029), 2.4027 (6.0820), 2.4672 (5.9831) and 1.5538 (1.1702), respectively. The positive S/CO values for P104 and S14P5 were significantly lower than others (*P* < 0.01, Fig. 4a). Such difference was also found in moderate patients (Fig. 4b). Moreover, epitope peptides S14P5 and S21P2 correlated positively with neutralizing antibody titre (Supplementary Figure 1, rs = 0.510, *P* < 0.01; rs = 0.227, *P* < 0.05), which was consistent with the experimental results reported by Poh et al. [11].

## Discussion

It has been reported that most COVID-19 patients were tested positively for antiviral IgG within 20 days after the onset of symptoms [12, 13], but a few patients did not show seroconversion [4]. The individual difference of seroconversion in COVID-19 patients was still to be considered. In this study, we enrolled 165 convalescent COVID-19 patients and collected their sera. Result of patients’ antibody test also showed a few patients did not seroconvert and the antibody level including RBD-IgG, RBD-Ab and neutralizing antibody of asymptomatic patients were lower than those of symptomatic patients. In order to clarify the relationship between the different type of antibodies, disease severity, age and sex, we further did a correlation analysis. We found RBD-IgG and RBD-Ab were moderately correlated with neutralizing antibody titre (r_s_ = 0.602, *P* < 0.01; r_s_ = 0.681, *P* < 0.01), which was consistent with other reports [14, 15]. The moderate correlation reflected the most of contribution to neutralization was from anti-RBD antibody, but the neutralization ability still required antibody from other regions to work together. We found there was only a weak correlation between antibody and age groups, perhaps due to the cases included in minor and the elder groups were much less than the young and middle-aged group that may weaken the trend. The disease severity was positively correlated with age groups (r_s_ = 0.542, *P* < 0.001). But Charitos I A et al. reported that old age was not an isolated risk factor for developing severe disease [16]. The underlying diseases and age-related immunity changes of the elderly contribute to increasing susceptibility to infections [17, 18].

Next, we identified B cell epitopes by using convalescent serum and S protein peptide library. Two B cell linear epitopes, P104 and P82, in the S2 subunit of the SARS-CoV-2 S protein were identified. They are located on the surface of the S2 subunit, and P104 located on the surface of the groove. P82 is completely contained in the S protein B cell linear epitope S21P2 sequence, that identified by Poh et al. [11]. Moreover, the sera tests showed that the positive reaction rate of P82 was lower than S21P2 (*P* < 0.01), but there was no significant difference between the positive IgG S/CO values. The sequence of P82 is 3 amino acids less than S21P2, which leads to the decrease in its positive reaction rate. It suggested that S21P2 is a more complete linear epitope of B cells. Therefore, P82 was no longer considered as a novel epitope in the subsequent analysis.

P104 is a new B cell linear epitope and hasn’t been reported. Epitope P104 had a low positive reaction rate in COVID-19 patients, which may be related to its spatial location. However, we found that the positive reaction rate of P104 in asymptomatic cases (36.7%) was higher than that in symptomatic cases (28.9%). Although the enhancement is not significant by statistical analysis, this epitope is the only one that showed higher detective rate for asymptomatic infections among the four. This phenomenon needs to be verified by more samples of asymptomatic infection cases, and its mechanism is worth studying in the future. P104 isn’t correlated with neutralizing antibody titre, perhaps because the location of P104 is far from RBD region. The binding of specific antibody with epitope P104 doesn’t interfere the viral infection mediated by RBD.

The epitope peptides S14P5 and S21P2 are both positive in more than 70% of COVID-19 convalescent sera. They also have recognition advantages for asymptomatic infections (> 50%). Our research verified the value of S14P5 and S21P2 of detection using more samples of COVID-19 convalescent sera than used in previous reports [11, 19], and firstly confirmed their potential for detecting asymptomatic infection. Also, our research confirmed the role of S14P5 specific antibody in virus neutralization via analyzing the correlation between IgG S/CO values of epitope with neutralizing antibody titre. S14P5 possesses possible ability of contributing to neutralizing effect may because of it located in subunit S1, which closed to RBD region [11]. As Röltgen K et al. reported, antibody could play neutralizing role through binding with non-RBD region, and low-affinity antibody might also have neutralizing activity [20].

Due to difference in individual immune responses and other reasons, detection positive rate of each epitope is quite different. And none of them could be recognized by over 80% in convalescent sera. Amrun et al. predicted by bioinformatics that combination of multiple peptides could enhance the sensitivity and specificity of detection to 100% [19]. In our research, we used real convalescent sera to verify that the positive rate will be enhanced after multiple epitopes combination, found that the positive rate of (S14P5 + S21P2 + P104) combination reached to 92.7% for total COVID-19 patients. This proved that the epitope peptides mixture was feasible as a detection antigen. With more epitopes be identified, some of them could be supplemented as an effective testing antigen in serum screening. It has been reported that the peptides-ELISA is effective in serological screening for virus infection by using human or animal serum, and the combination of epitopes can improve the specificity and sensitivity of detection [9, 12]. However, due to the lack of the serum samples from SARS-CoV, MERS-CoV survivors or donors confirmed to have been infected with common coronaviruses, we do not know the cross-reactivity of the peptides with the other human coronavirus. Therefore, it needs to consider the possible cross-reactivity when applying.

With COVID-19 spreading around the world, the specificity of detection method is particularly important. Therefore, many studies have proposed optimized methods, including nucleic acid detecting and antibody screening [21–23], to meet the requirements of high-throughput, rapid and accurate detection. At present, the method of confirming SARS-CoV-2 infection is still mainly based on the detection of viral nucleic acid. At the same time, more and more studies have shown that serological test also has excellent specificity for identifying COVID-19 patients [24], especially the early asymptomatic infections [3]. Epidemiological research showed that the number of asymptomatic infections was underestimated in many countries [25–27], which have an impact on the prevention and control of the epidemic. Selection of specific epitope peptides as detection antigen, with their simple primary structure, reduces the risk of cross-reaction by memory B cells to the other human coronavirus in vivo [28]. And the usage of peptides also promotes production efficiency. The combination of RNA detection and serological test can significantly improve the sensitivity [29]. As a supplemental tool to nucleic acid detection, antibody test assists in the confirmation COVID-19 patients and reduces the exposure risk of repeated sampling.

At present, the COVID-19 vaccines have been vaccinated in many countries [30–32]. The clinical trial results showed that the vaccine could induce seroconversion and specific antibody in subjects [33], which had a similar antibody profile with COVID-19 patients’. Since the antibody level of convalescent patients began to decline in 2–3 months after infection [34], it is necessary to keep monitoring the antibody level in the vaccinated population to evaluate the protection of the vaccines.

Recently, more SARS-CoV-2 S protein B cell linear epitopes have been identified, and improved SARS-CoV-2 epitope distribution information [35, 36]. Our research data together with others will play an important role in promoting the understanding of the immune mechanism of the virus, vaccine design and evaluation and antibody tests.

## Methods

### Information regarding discharged COVID-19 patients and sample collection

From March 5, 2020 to May 12, 2020, 165 discharged COVID-19 patients, including those without symptoms, in Shenzhen were enrolled in this study. The convalescent sera of patients were collected within 14 days after discharge. The 165 discharged COVID-19 patients were treated at Shenzhen Third People’s Hospital and reached the discharge standard for COVID-19 patients, as follows: body temperature returned to normal for more than 3 consecutive days; respiratory symptoms improved significantly; lung imaging showed significant improvement in acute exudative lesions; and twice consecutive nucleic acid tests showed negative were obtained using sputum, nasopharyngeal swabs and other respiratory tract samples (at least 24 h apart). According to the Novel Coronavirus Pneumonia Diagnosis and Treatment Plan (Provisional 7th Edition) promulgated by the National Health Commission of the People’s Republic of China [37], the clinical classification criteria for COVID-19 patients are as follows: (1) Mild patients have mild clinical symptoms and no pneumonia manifestations in imaging. (2) Moderate patients have fever, respiratory symptoms and others, imaging findings of pneumonia. (3) Severe adults meet one of the followings: ① Shortness of breath, respiratory rate ≥ 30 times/min; ② In resting state, the oxygen saturation ≤ 93%; ③ Partial pressure of oxygen (PaO2) / oxygen concentration (fraction of inspiration oxygen, FiO2) ≤300 mmHg (1 mmHg = 0.133 kPa). In areas with a high altitude (over 1000 m above sea level), PaO2/FiO2 should be corrected according to the following formula: PaO2/FiO2 × [atmospheric pressure (mmHg) / 760]. Lung imaging shows that the lesions progress significantly within 24 to 48 h > 50% are treated as severe. Severe children meet one of the followings: ① Shortness of breath (< 2 months old, respiratory rate ≥ 60 times/min; 2–12 months old, respiratory rate ≥ 50 times/min; 1 to 5 years old, respiratory rate ≥ 40 times/min. > 5 years old, respiratory rate ≥ 30 times/min), except for the effects of fever and crying; ② In resting state, finger oxygen saturation ≤ 92%; ③Assisted breathing (groaning, nasal flapping, three-concave sign), cyanosis, intermittent apnea; ④Drowsiness, convulsions; ⑤ Refusing to feed or feeding difficulties, with signs of dehydration. (4) Critical patients meet one of the following conditions: ① Respiratory failure and the need for mechanical ventilation; ② Shock; ③ Patients with other organ failure should be treated in ICU. Asymptomatic infection is defined as a positive throat swab nucleic acid test but no symptoms related to SARS-CoV-2 infection. For negative controls, serum was collected from twenty healthy people [without SARS-CoV-2 infection and with negative human immunodeficiency virus (HIV), hepatitis C virus (HCV), and syphilis serological test results]. The research process was conducted in strict accordance with ethical requirements and was reviewed by the Ethics Committee of the Shenzhen Centre for Disease Control and Prevention of Guangdong Province (QS2020070048).

### Antibody detection

Chemiluminescence microparticle immunoassay (CMIA) technology (Caris 200 automatic chemiluminescence instrument, Beijing Wantai Biotech, China) was used to detect cutoff index (COI) values (sample detection value/cutoff value) for total antibody (Ab) and IgG levels against SARS-CoV-2 S-RBD in serum samples.

A SARS-CoV-2 virus isolate (20SF014/vero-E6/3) and Vero-E6 cells were used for the determination of serum neutralizing antibody titre. Vero-E6 cells were seeded in 96-wells plates at 1 ~ 2 × 10^4^ cells/100 μL and cultured for 12 h (37 °C, 5% CO_2_). After the serum was heat-inactivated at 56 °C for 30 min, serial dilutions of 1:4, 1:16, 1:64, 1:256 and 1:1024 were generated. The SARS-CoV-2 virus was incubated with serum at a titre of 104.67 TCID50/50 μL for 2 h. Vero-E6 cells were inoculated with the 100 μL mixture of virus and serum. Virus positive controls were prepared for virus titre of 100 TCID50/50 μL, 10 TCID50/50 μL, 1 TCID50/50 μL, and 0.1 TCID50/50 μL and incubated at the same time as the samples. When the TCID50/50 μL virus control wells showed a complete cytopathic effect, the highest dilution of the test serum at which half of the cells had no cytopathic effect was recorded as the neutralizing antibody titre of the serum sample. If the titre exceeded 1:4, it considered to be positive.

### Peptide library

The amino acid sequence of the SARS-CoV-2 S protein (NCBI access number: YP_009724390.1) was used to design and synthesize peptides. Peptides with a length of 15 amino acids were synthesized according to the amino acid sequence, with adjacent peptides overlapping by 5 amino acids. A total of 127 peptides (P1 ~ P127) were obtained, covering the full length of the S protein, synthesized by GenScript Biotech Corporation (Nanjing, China). The peptides were divided into 13 groups (G1 ~ G13) in the order of amino acid position, with each group containing 10 or 7 consecutive peptides (Supplementary Table 2).

### B cell linear epitope screening process

First, each peptide group (G1 ~ G13) was used to separately coat an ELISA 96-wells plate. Convalescent sera from twenty discharged COVID-19 patients, including fourteen moderate patients, three mild patients and three asymptomatic infections, were randomly selected and mixed as test sera for indirect ELISA. Meanwhile, twenty serum samples from healthy people were pooled to a mixed sample as negative control. The peptide groups with positive reactions were selected. Each peptide in the positive peptide group was then used to separately coat a 96-wells plate, and indirect ELISA detection was subsequently performed with serum to determine peptides generating a positive reaction (Supplementary Figure 2).

### Indirect ELISA

ELISA 96-wells plates (Corning, #42592, USA) were coated overnight at 4 °C with 100 μL mixed peptides (2.5 μg/mL of each peptide) or 0.2 μg/mL RBD (CUSABIO, #CSB-MP3324GMY1b1, China) as positive antigen control. Following coating, the plates were washed with 300 μL wash buffer (PBS containing 0.05% Twenn-20, Sangon Biotech, China). Blocking with 300 μL /well of blocking solution (PBS containing 0.1%Tween-20 and 5% skimmed milk powder, Sangon Biotech, China) for 2 h. After washing, wells were covered with 100 μL heat-inactivated (56 °C for 30 min) test sera or negative sera diluted at 1:80 in blocking solution and were incubated for 1 h at 37 °C. Each sample was tested in duplicate. After incubation, the plates were washed and added 100 μL /well of solution (PBS containing 0.05% Tween-20 and 1% BSA, Sangon Biotech, China) containing rabbit anti-human IgG HRP conjugate (abcam, #6759, UK) that diluted at 1:130000, and were incubated for 1 h at 37 °C. After incubation, the plates were washed and added 100 μL TMB (Multi Science, #EK0011, China) to the wells. Plates were incubated in the dark at room temperature for 25 min. The reaction was stopped with 100 μL /well of stop solution (Multi Science, #EK0011, China). Determined the absorbance at 450 nm and 630 nm (Tecan Infinite M200, Switzerland), and calculated the value of OD450 minus OD630 according to the instruction. Cutoff values were determined as mean + 3 × standard deviation (SD) of negative sera [38, 39]. Positive reaction was regarded as the S/CO value (sample average OD450 nm–630 nm value/cutoff value) of test sera was greater than 1.

### Serological detection of B cell linear epitopes

The epitope peptides S14P5 and S21P2 were synthesized by Sangon Biotech (Shanghai, China). Four B cell linear epitopes (S21P2, S14P5, P82, and P104) were used to coat 96-wells plates, separately, and each COVID-19 convalescent serum sample was tested by indirect ELISA. Each sample was tested in duplicate. The pooled healthy people sera used as negative sera to calculate the cutoff value and then to calculate the S/CO value. Indirect ELISA was performed as described above.

### Data analysis

We used SPSS Statistics 21 and GraphPad Prism 8.0 for statistical analyses and plotting. The 3D structure of the protein was examined using an NCBI online tool. The chi-square test was applied to analyse the positivity rates of epitope peptides. The Kruskal-Wallis *H* test was carried out to assess differences in serum RBD-IgG, RBD-Ab, neutralizing antibody titres and epitope peptide antibody S/CO values. The Spearman correlation test was used for correlation analyses. When the *P* value was less than 0.05, the difference considered to be statistically significant, whereby * represents *P* < 0.05, ** represents *P* < 0.01, and *** represents *P* < 0.001.

## Conclusion

In conclusion, this study identified a B cell linear epitope P104 that located in the S protein of SARS-CoV-2. Compared in symptomatic patients, P104 showed slight detection advantage in asymptomatic infections. Our research confirmed the combination of several specific epitope peptides could significantly increase the positive detection rates. It provided experimental data support for the application of epitope peptide-ELISA tested in COVID-19 patients. At the same time, the results also showed the contribution of non-RBD epitope peptide in antibody detection.

## Supplementary Information

**Additional file 1. **Further data are available as Supplementary Material. **Table S1.** Sequence and position of peptide. **Table S2.** Peptides sequence of overlapping peptide pool. **Figure S1**. The correlation analyses of neutralizing antibody titre with antibody level of S14P5 and S21P2. **Figure S2.** Program for screening of positive peptides.

## Data Availability

The date supporting findings in this study are available within the paper. Other data can be obtained from corresponding author upon reasonable request.

## Abbreviations

ELISA: Enzyme linked immunosorbent assay
3D structure: Three-dimensional structure
SARS-CoV: Severe acute respiratory syndrome coronavirus
MERS –CoV: Middle east respiratory syndrome coronavirus
RNA: Ribonucleic acid
ICU: Intensive Care Unit
TCID50: Median tissue culture infective dose
PBS: Phosphate buffer saline
BSA: Bovine Serum Albumin
HRP: Horseradish Peroxidase
TMB: Tetramethylbenzidine
