# Supplementary material for: A newly identified linear epitope on non-RBD region of SARS-CoV-2 spike protein improves the serological detection rate of COVID-19 patients
Source: BMC Microbiol. 2021 Jun 26;21:194. doi: 10.1186/s12866-021-02241-y (PMC8234764; doi:10.1186/s12866-021-02241-y)
Supplement: Supplementary file 1 — Additional file 1. Further data are available as Supplementary Material. Table S1. Sequence and position of peptide. Table S2. Peptides sequence of overlapping peptide pool. Figure S1. The correlation analyses of neutralizing antibody titre with antibody level of S14P5 and S21P2. Figure S2. Program for screening of positive peptides. [file 12866_2021_2241_MOESM1_ESM.docx]

A newly identified linear epitope on non-RBD region of SARS-CoV-2 spike protein improves the serological detection rate of COVID-19 patients

Yunwen Zhang^1,2,3#^, ZhengrongYang^3#^, Sicheng Tian^1,2#^, Baisheng Li^4#^, Tiejian Feng^3^, Jianfan He^3^, Min Jiang^3^, Xiujuan Tang^3^, Shujiang Mei^3^, Hao Li^3^, Yifan Zhong^3^, Guilian Li^3^, Mingyuan Tang^1,2^, Sijing Liu^1,2^, Tian Tang^1,2^, Chuan Wang^1,2*^, Xiaohui Wang^3*^

^1^ West China School of Public Health and West China Fourth Hospital, Sichuan University, Chengdu, P. R. China

^2^ Food Safety Monitoring and Risk Assessment Key Laboratory of Sichuan Province, Department of Public Health Laboratory Sciences, West China School of Public Health, Sichuan University, Chengdu, China

^3^Shenzhen Center for Disease Control and Prevention, Shenzhen, China

^4^Guangdong Provincial Center for Disease Control and Prevention, Guangdong, China

*Correspondence:

Xiaohui Wang

[wangxh@szcdc.net](mailto:wangxh@szcdc.net)

Chuan Wang

[wangchuan@scu.edu.cn](mailto:wangchuan@scu.edu.cn)

#These authors contributed equally to this work.

Supplemental material

**Table. S1** Sequence and position of peptide

| **peptide** | **sequence** | **position** |
| --- | --- | --- |
| P82 | KPSKRSFIEDLLFNK | 811-825^a^ |
| P104 | ECVLGQSKRVDFCGK | 1031-1045^a^ |
| S14P5 | TESNKKFLPFQQFGRDIA | 553-570^a^ |
| S21P2 | PSKPSKRSFIEDLLFNKV | 809-826^a^ |

^a^: indicated amino acid position of SARS-CoV-2 spike protein (amino acid sequence of spike protein accession number：YP_009724390.1)

**Table. S2** Peptides sequence of overlapping peptide pool

| **Group** | **Peptide** | **Sequence** | **Group** | **Peptide** | **Sequence** |
| --- | --- | --- | --- | --- | --- |
| G1 | P1 | MFVFLVLLPLVSSQC | G2 | P11 | IRGWIFGTTLDSKTQ |
|  | P2 | VSSQCVNLTTRTQLP |  | P12 | DSKTQSLLIVNNATN |
|  | P3 | RTQLPPAYTNSFTRG |  | P13 | NNATNVVIKVCEFQF |
|  | P4 | \| SFTRGVYYPDKVFRS \| \| --- \| |  | P14 | CEFQFCNDPFLGVYY |
|  | P5 | KVFRSSVLHSTQDLF |  | P15 | LGVYYHKNNKSWMES |
|  | P6 | TQDLFLPFFSNVTWF |  | P16 | SWMESEFRVYSSANN |
|  | P7 | NVTWFHAIHVSGTNG |  | P17 | SSANNCTFEYVSQPF |
|  | P8 | SGTNGTKRFDNPVLP |  | P18 | VSQPFLMDLEGKQGN |
|  | P9 | NPVLPFNDGVYFAST |  | P19 | GKQGNFKNLREFVFK |
|  | P10 | YFASTEKSNIIRGWI |  | P20 | EFVFKNIDGYFKIYS |
| G3 | P21 | FKIYSKHTPINLVRD | G4 | P31 | CTLKSFTVEKGIYQT |
|  | P22 | NLVRDLPQGFSALEP |  | P32 | GIYQTSNFRVQPTES |
|  | P23 | SALEPLVDLPIGINI |  | P33 | QPTESIVRFPNITNL |
|  | P24 | IGINITRFQTLLALH |  | P34 | NITNLCPFGEVFNAT |
|  | P25 | LLALHRSYLTPGDSS |  | P35 | VFNATRFASVYAWNR |
|  | P26 | PGDSSSGWTAGAAAY |  | P36 | YAWNRKRISNCVADY |
|  | P27 | GAAAYYVGYLQPRTF |  | P37 | CVADYSVLYNSASFS |
|  | P28 | QPRTFLLKYNENGTI |  | P38 | SASFSTFKCYGVSPT |
|  | P29 | ENGTITDAVDCALDP |  | P39 | GVSPTKLNDLCFTNV |
|  | P30 | CALDPLSETKCTLKS |  | P40 | CFTNVYADSFVIRGD |
| G5 | P41 | VIRGDEVRQIAPGQT | G6 | P51 | NGVGYQPYRVVVLSF |
|  | P42 | APGQTGKIADYNYKL |  | P52 | VVLSFELLHAPATVC |
|  | P43 | YNYKLPDDFTGCVIA |  | P53 | PATVCGPKKSTNLVK |
|  | P44 | GCVIAWNSNNLDSKV |  | P54 | TNLVKNKCVNFNFNG |
|  | P45 | LDSKVGGNYNYLYRL |  | P55 | FNFNGLTGTGVLTES |
|  | P46 | YLYRLFRKSNLKPFE |  | P56 | VLTESNKKFLPFQQF |
|  | P47 | LKPFERDISTEIYQA |  | P57 | PFQQFGRDIADTTDA |
|  | P48 | EIYQAGSTPCNGVEG |  | P58 | DTTDAVRDPQTLEIL |
|  | P49 | NGVEGFNCYFPLQSY |  | P59 | TLEILDITPCSFGGV |
|  | P50 | PLQSYGFQPTNGVGY |  | P60 | SFGGVSVITPGTNTS |
| G7 | P61 | GTNTSNQVAVLYQDV | G8 | P71 | AENSVAYSNNSIAIP |
|  | P62 | LYQDVNCTEVPVAIH |  | P72 | SIAIPTNFTISVTTE |
|  | P63 | PVAIHADQLTPTWRV |  | P73 | SVTTEILPVSMTKTS |
|  | P64 | PTWRVYSTGSNVFQT |  | P74 | MTKTSVDCTMYICGD |
|  | P65 | NVFQTRAGCLIGAEH |  | P75 | YICGDSTECSNLLLQ |
|  | P66 | IGAEHVNNSYECDIP |  | P76 | NLLLQYGSFCTQLNR |
|  | P67 | ECDIPIGAGICASYQ |  | P77 | TQLNRALTGIAVEQD |
|  | P68 | CASYQTQTNSPRRAR |  | P78 | AVEQDKNTQEVFAQV |
|  | P69 | PRRARSVASQSIIAY |  | P79 | VFAQVKQIYKTPPIK |
|  | P70 | SIIAYTMSLGAENSV |  | P80 | TPPIKDFGGFNFSQI |
| G9 | P81 | NFSQILPDPSKPSKR | G10 | P91 | QMAYRFNGIGVTQNV |
|  | P82 | KPSKRSFIEDLLFNK |  | P92 | VTQNVLYENQKLIAN |
|  | P83 | LLFNKVTLADAGFIK |  | P93 | KLIANQFNSAIGKIQ |
|  | P84 | AGFIKQYGDCLGDIA |  | P94 | IGKIQDSLSSTASAL |
|  | P85 | LGDIAARDLICAQKF |  | P95 | TASALGKLQDVVNQN |
|  | P86 | CAQKFNGLTVLPPLL |  | P96 | VVNQNAQALNTLVKQ |
|  | P87 | LPPLLTDEMIAQYTS |  | P97 | TLVKQLSSNFGAISS |
|  | P88 | AQYTSALLAGTITSG |  | P98 | GAISSVLNDILSRLD |
|  | P89 | TITSGWTFGAGAALQ |  | P99 | LSRLDKVEAEVQIDR |
|  | P90 | GAALQIPFAMQMAYR |  | P100 | VQIDRLITGRLQSLQ |
| G11 | P101 | LQSLQTYVTQQLIRA | G12 | P111 | HWFVTQRNFYEPQII |
|  | P102 | QLIRAAEIRASANLA |  | P112 | EPQIITTDNTFVSGN |
|  | P103 | SANLAATKMSECVLG |  | P113 | FVSGNCDVVIGIVNN |
|  | P104 | ECVLGQSKRVDFCGK |  | P114 | GIVNNTVYDPLQPEL |
|  | P105 | DFCGKGYHLMSFPQS |  | P115 | LQPELDSFKEELDKY |
|  | P106 | SFPQSAPHGVVFLHV |  | P116 | ELDKYFKNHTSPDVD |
|  | P107 | VFLHVTYVPAQEKNF |  | P117 | SPDVDLGDISGINAS |
|  | P108 | QEKNFTTAPAICHDG |  | P118 | GINASVVNIQKEIDR |
|  | P109 | ICHDGKAHFPREGVF |  | P119 | KEIDRLNEVAKNLNE |
|  | P110 | REGVFVSNGTHWFVT |  | P120 | KNLNESLIDLQELGK |
| G13 | P121 | QELGKYEQYIKWPWY | | | |
|  | P122 | KWPWYIWLGFIAGLI | | | |
|  | P123 | IAGLIAIVMVTIMLC | | | |
|  | P124 | TIMLCCMTSCCSCLK | | | |
|  | P125 | CSCLKGCCSCGSCCK | | | |
|  | P126 | GSCCKFDEDDSEPVL | | | |
|  | P127 | SEPVLKGVKLHYT | | | |


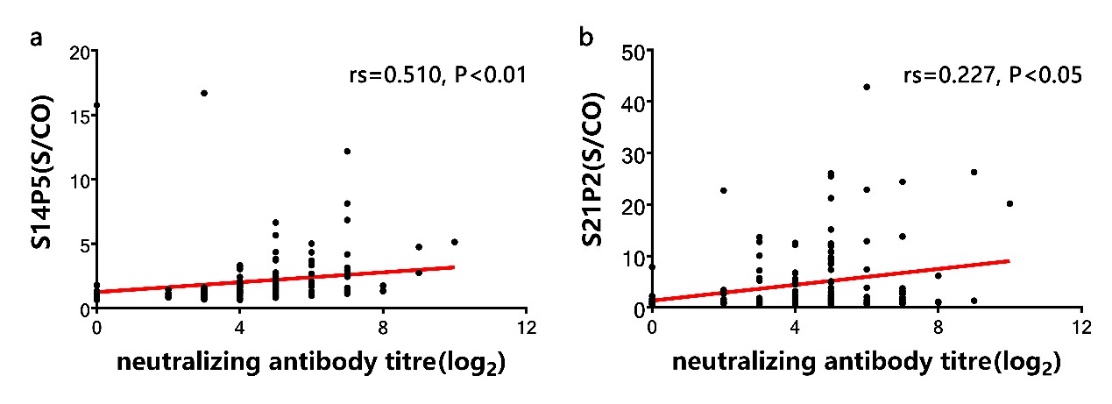


**Fig. S1** The correlation analyses of neutralizing antibody titre with antibody level of S14P5 and S21P2. The figure showed scatter plots and Spearman correlation coefficients, and the red line was trend line.


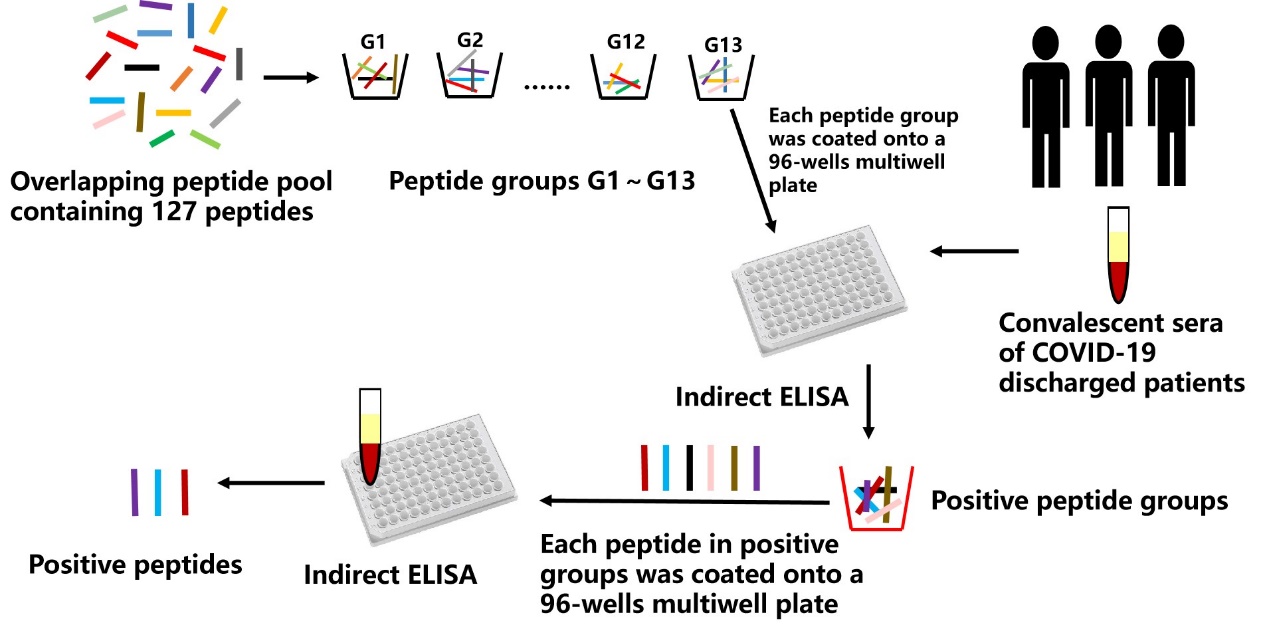


**Fig. S2** Program for screening of positive peptides.
